# The ontogenesis of narrative: from moving to meaning

**DOI:** 10.3389/fpsyg.2015.01157

**Published:** 2015-09-02

**Authors:** Jonathan T. Delafield-Butt, Colwyn Trevarthen

**Affiliations:** ^1^Early Years, School of Education, Faculty of Humanities and Social Sciences, University of StrathclydeGlasgow, UK; ^2^School of Philosophy, Psychology and Language Sciences, College of Humanities and Social Sciences, The University of EdinburghUK

**Keywords:** narrative, motor origins, ontogenesis, embodied meaning-making, intersubjectivity theory, communication, intentionality

## Abstract

Narrative, the creation of imaginative projects and experiences displayed in expressions of movement and voice, is how human cooperative understanding grows. Human understanding places the character and qualities of objects and events of interest within stories that portray intentions, feelings, and ambitions, and how one cares about them. Understanding the development of narrative is therefore essential for understanding the development of human intelligence, but its early origins are obscure. We identify the origins of narrative in the innate sensorimotor intelligence of a hypermobile human body and trace the ontogenesis of narrative form from its earliest expression in movement. Intelligent planning, with self-awareness, is evident in the gestures and motor expressions of the mid-gestation fetus. After birth, single intentions become serially organized into projects with increasingly ambitious distal goals and social meaning. The infant imitates others’ actions in shared tasks, learns conventional cultural practices, and adapts his own inventions, then names topics of interest. Through every stage, in simple intentions of fetal movement, in social imitations of the neonate, in early proto-conversations and collaborative play of infants and talk of children and adults, the narrative form of creative agency with it four-part structure of ‘introduction,’ ‘development,’ ‘climax,’ and ‘resolution’ is present. We conclude that shared rituals of culture and practical techniques develop from a fundamental psycho-motor structure with its basic, vital impulses for action and generative process of thought-in-action that express an integrated, imaginative, and sociable Self. This basic structure is evident before birth and invariant in form throughout life. Serial organization of single, non-verbal actions into complex projects of expressive and explorative sense-making become conventional meanings and explanations with propositional narrative power. Understanding the root of narrative in embodied meaning-making in this way is important for practical work in therapy and education, and for advancing philosophy and neuroscience.

## Introduction: The Primary Motives for Stories of Common Sense

“It were easy to show, that the fine arts of the musician, the painter, the actor, and the orator, so far as they are expressive… are nothing else but the language of nature, which we brought into the world with us, but have unlearned by disuse and so find the greatest difficulty in recovering it. … That without a natural knowledge of the connection between these (natural) signs and the things signified by them, language could never have been invented and established among men; and, that the fine arts are all founded upon this connection, which we may call the natural language of mankind. … It is by natural signs chiefly that we give force and energy to language; and the less language has of them, it is the less expressive and persuasive.”

(Reid, 1764/1997, p. 53, 59, 106–107).

“Narrative structure is even inherent in the praxis of social interaction before it achieves linguistic expression.”

(Bruner, 1990, p. 77)

### The Human Impulse for Meaning, and its Early Cultivation

Young children, in their families, before school, show how our stories of life begin in artful invention (Bullowa, 1979; Trevarthen and Delafield-Butt, 2015). They grasp knowledge purposefully and creatively, encouraged by the convivial imagination of parents, family, and friends (Donaldson, 1978; Halliday, 1978; Bruner, 1996; Rogoff, 2003; Legerstee, 2005; Reddy, 2008, 2012; Mazokopaki and Kugiumutzakis, 2009; Frank and Trevarthen, 2012; Trevarthen, 2013; Trevarthen et al., 2014). The meaning of their natural ‘common sense’ grows with an intuitive ‘logic of action and interaction’ (Lashley, 1951). Events invented or recalled are shared in the dramatic time-based performances that give rise to film, theater, music, and dance (Turner, 1982; Bjørkvold, 1992; Dissanayake, 2000; Stern, 2000; Gratier and Trevarthen, 2008; Malloch and Trevarthen, 2009).

This human imagination of a young child has a further ambition to identify life events out of the present time of action, learning how to refer to them in abstract, symbolic terms (Donaldson, 1992; Osborne, 2009). A scientific focus on this cognitive abstraction of meaning, and especially on the record of experience in text, attracts attention away from the present moment into a timeless world of ideas (Brandt, 2009). But our intelligence grows in shared story-making. As we learn words and how to use them in syntactic series to tell stories of imaginary agents, the meaning is always in terms of the motivations of these characters within their present world, the effects of their movements on other objects and persons, and ultimately the lasting, vital result or ‘fate’ of those concerned (Bruner, 2002).

The impulses of even the most sophisticated narrative derive from the common ‘vitality dynamics’ of play in infancy (Stern, 2010), with characteristic phases of arousal and moments of focussed intensity (Amighi et al., 1999; Damasio, 1999; Trevarthen et al., 2011; Trevarthen and Delafield-Butt, 2013a), giving feeling to perceptions of outer things by projection of symptoms of inner autonomic activity – heart rate changes, breathing, flight-or-fight response, all displayed and shared in specially adapted expressive movements to convey felt meaning in ‘natural language’ (Porges, 1997; Porges and Furman, 2011). Within these dynamic emotional events, relations between objects and participants, their properties, motivation and character, can become placed and named in ‘artificial,’ learned and conventional language.

Narrative consciousness, with its cognitive content, rather than conceived as a product of conceptual verbal thinking, can be defined as the organizing life principle of human cognition (Bruner, 1990) animated by a primary emotional consciousness (Panksepp, 2005) in social events of meaning-making (De Jaegher et al., 2010). It is by making and telling affected stories that we represent the importance to ourselves of other persons’ presence and actions, the properties of objects, how persons and objects relate to each other, and to one’s own well-being in awareness of activity. The assumptions, understandings, and knowledge of science, law, politics, history, and religion all depend on the developmental construction, co-construction, and re-telling of narratives, with or without words (Halliday, 1975, 1978; Bråten, 2009).

Notwithstanding the evident truth of the powers of human imagination and their sharing made in movement, it is not clear, in the science of language development, how these narratives of meaning-making, ubiquitous in human life and its intelligence, first appear in development (Cobley, 2014, p. 1–27). Their ontogenesis before the development of words and language remain largely unknown (Dautenhahn, 2002). Understanding the psychobiological source of living narrative requires information concerning the creation of the intelligence of an integrated, affectively conscious agent capable of anticipating the outcomes of processes of movement, and their vital importance (Turner, 1996).

In this paper, we trace the origins of narrative meaning-making back to the earliest explorations of action by the human fetus, *in utero*. We note how impulses for making sense of the body in its world develop and learn, both before and after birth in solitary and in social engagements, and how they become elaborated in more complex compositions (Pezzulo, 2011; Delafield-Butt and Gangopadhyay, 2013). Individual projects are generated and shared in intersubjective episodes that make up the collaborative narratives of sound and gesture in pre-verbal proto-conversations (Bateson, 1979; Gratier and Trevarthen, 2008; Trevarthen and Delafield-Butt, 2013a). Later with articulate language, conversation develops with signs and symbols to represent events, feelings, intentions, and objects. So the stories become more specialized and defined (Delafield-Butt and Trevarthen, 2013).

### The Animation of a Narrative

At all stages of a life of learning, from the playful displays of conviviality of early childhood to the development of sophisticated works of art, philosophy, or science, narrative activities are generated and sequenced in a chronobiology of ‘vital time’ (Trevarthen, 2008, 2015; Osborne, 2009; Stern, 2010). There is a remarkable consistency in the performance and emotional regulation of narratives in development at every stage: in simple self-produced intentions of fetal movement, in intersubjective inventions of imitation with a neonate, in early proto-conversations and collaborative play of older infants, in inventive talk of toddlers and adults, and in effective practice of teaching. Each episode of creative agency, or story, exhibits four states of arousal that regulate the flow of interest and the pleasure of engagement. These are: ‘introduction,’ ‘development,’ ‘climax,’ and ‘resolution.’ To explain this we must turn attention to the processes that generate and regulate animal movements with prospective sensory control.

### Purpose and Feeling in Movement

Animal movements are unlike the motions of inanimate objects, because they are self-generated and purposeful, guided by an anticipation of specific sensual consequences by selective orientation of receptors and aimed actions. This primary consciousness, or ‘*with-knowing-ness*,’ is generated as muscle action, which is controlled by proprioception or self-sensing through measures of time by an integrative nervous system (Sherrington, 1906; Bernstein, 1967; Llinás, 2001; Buzsáki, 2006; Panksepp, 2011). The execution of a simple act, such as a reach-to-touch an object, or a turn of the head and eyes to fixate a point with sight, is prospectively organized by concerted and precisely sequenced action of many muscles moving several body parts with anticipation of a particular pattern of sensory consequences. It is intentional, and goal-directed (Trevarthen, 1978; von Hofsten, 2007; Lee, 2009; Delafield-Butt and Gangopadhyay, 2013). It exhibits initiation toward the goal, progression with fast rhythmic timing of corrective maneuvers keeping the movement on track, and final climactic contact with its object, as a unit of meaning-making that tests and confirms a knowledge of expectations. When completed, the organism has placed itself in a new set of relations with its environment, and can prepare to exploit awareness of this, together with its past that is now held in memory, for the next project. There exists a simple intelligence within animal movement, a basic motor logic that strives to preserve and expand the vitality of the organism in its relations with its perceived objectives.

All self-generated animal movements, even the simplest and most primitive, are future-oriented. Their muscle activity conforms to a coordinating ‘motor image’ or ‘schema’ of purpose (Bernstein, 1967; Jeannerod, 1988; Lee, 2009). ‘Unconditioned’ reflex reactions to stimuli only occur in emergency, for immediate correction of aim, or for defense and escape. The impulse to move is planned with adaptation to sustain or benefit the vitality of the integrated self-conscious organism (Goodrich, 2010), and this becomes the foundation for acts-of-meaning in social communication, cooperative action, and the building of affective relations (Sebeok, 1972, 1994; Trevarthen, 1978, 1986a). In a well-conceived sequence of acts, an ultimate goal determines the pattern of the whole project by distributed control of a hierarchy of movements (Lashley, 1951; Jeannerod, 1988), awareness of which may be transmitted to others who are observers, or an audience, as a moving story.

The first intentional consciousness in animal life may be regarded as pre-conceptual and pre-reflective, without separation into a cognitive representation of an outer reality (Delafield-Butt and Gangopadhyay, 2013). These basic purposes, or ‘intentions-in-action’ (Searle, 1983), are evaluated by inner reference to states of ‘primary affective consciousness’ located in brainstem integrative systems, which do not require a functioning cerebral cortex (Panksepp, 2011; Panksepp and Biven, 2012; Solms and Panksepp, 2012). Their actions, with perceptual information and records of detail in memory transmitted from the cerebral neocortex, are integrated with precision by the time-keeping powers of the cerebellum responsible for whole-body cognition (Koziol et al., 2013; Llinás and Negrello, 2015). This theory of the psychobiology of animal agency constitutes a drastic revision of the priority that the theory of cognitive neuroscience has generally given to cortical discriminations and their articulation in language, affirming that the brainstem, while *anatomically sub-cortical* is *functionally supra-cortical* (Merker, 2007).

A brainstem-based consciousness that is perceptive, affective, and anticipating future contingencies of intentional action in a whole-body-related action-space is developed in infant humans as an adaptable mental agency that is intelligent, purposive, and a generator of meaning (Baldwin, 1895; Piaget, 1953, 1954). “Human language and thought can be regarded as… deriving from neuroanatomical systems that generate overt motor responses to environmental challenges and opportunities” (Lieberman, 2002, p. 158), the basis of which are brainstem sensorimotor and affective integrative systems (Merker, 2007; Panksepp and Biven, 2012).

### Development of Human Intelligence and Common Sense

The first pre-reflexive, pre-conceptual acts of ‘meaning-making’ of a human person develop from the spontaneous, self-generated ‘writhing’ movements of the integrated organism evident in the seventh week of gestation, when the embryo is only 2 cm in length (Lüchinger et al., 2008; Einspieler et al., 2012). By 8 weeks of gestation, displacements of the limbs and thorax with partial rotations of the head are produced in well-formed ‘general movements,’ but are not yet discrete, nor focussed on external goals (de Vries et al., 1984; Lüchinger et al., 2008; Piontelli, 2010). But by 10 weeks gestational age, fetal arm movements become differentiated from general body movement and hand movements are directed to parts of the body, especially to the face and head (Piontelli, 2010), giving the first indication of a motivation for developing a primary awareness of the Self.

Purposeful actions with the whole body or by separate actions of parts, such as the hands, develop in the second trimester (Zoia et al., 2007, 2013; Piontelli, 2010). These actions depend on both an internal proprioceptive sense of the body in motion and on ex-proprioceptive touch picking up information of changing relations with external objects, such as the wall of the mother’s uterus, or the body of a twin (Castiello et al., 2010), or from hearing the mother’s voice (DeCasper and Spence, 1986). These early intentional acts motivate an extension of the imaginative use of the body into the future, guided by prospective perceptual awareness which is beginning to inform a memory of consequences (Trevarthen, 1984; Jeannerod, 2006; Reissland et al., 2013a). Movements are assembled into more purposeful complexes, such as bicycling the legs against the uterine wall coincident with a trunk rotation, causing the fetus to turn over, or a reach and grasp directed to the umbilical cord (Piontelli, 2010).

Recent studies with four-dimensional ultrasound find not only that facial movements made in the last trimester of fetal life may be organized to express displeasure or smiles of enjoyment in affective responses to different stimuli, but that mouth movements show adaptations toward both execution of speech movements and imitative ‘mirroring’ of sounds of speech (Reissland et al., 2011, 2013b). The expressive movements of both kinds, as well as self-touching movements of the hands, show asymmetries that can be related to the cerebral asymmetries of neocortical function that develop in early childhood, and that become particularly important for the learning of language (Reissland et al., 2014, Reissland et al., 2015).

After birth this conscious human agency seeks not only to discover further knowledge generated through forms of activity of the Self, as Piaget (1953, 1954) described, but to share vitality in what comes to be identified in adult meaning-making as ‘narrative’ in language, regulated by conceptions of Self-Other consciousness (Legerstee, 2005). The appetite for sharing a narrative is clearly demonstrated by the powers of infants to both imitate expressive movements and collaborate in their sequencing to ‘tell a story,’ even within hours of birth (Trevarthen and Delafield-Butt, 2013a, 2015; Kugiumutzakis and Trevarthen, 2015).

The template or program for picking-up information and making sense of the world is formed from the basic, future-oriented nature of movement. The individual-as-agent extends forward in time through movement, self-generating a world of actions and contingent perceptual responses. These responses are appraised as a threat or benefit to the vitality of the organism, giving a self-related affective valence to each act and it consequences. *Meaning* is achieved by moving with assimilation of contingent sensory stimulation, and with emotion, not by a passive stimulus-response mechanism of mind, but by the living creativity of an embodied, psycho-physical organism. The fundamental narrative form as a self-generated experience, with the four-part structure of its vitality, (i) ‘introduction,’ (ii) ‘development,’ (iii) ‘climax,’ and (iv) ‘resolution,’ is evident in the simplest elementary action of the agent to more complex projects of actions that extend over greater domains of time and space (**Table 1**).

**Table 1:** Units of solitary and social sensorimotor intentionality.

Level	Unit type	Description	Temporal range
Primary, solitary	Action unit	A single continuous profile of velocity to a goal, obeying ‘tau’ dynamics for ‘gap closure’ e.g., an arm movement to a point in body-space or to contact an object.	200–1200 ms
Primary, social	Shared action unit	A single continuous movement to a goal made in shared attention with another – to sustain mutual gaze, to shared a gesture, or to achieve harmonic concordance in vocal exchange	
Secondary, solitary	Immediate, proximal project -sequence of actions	Coordination and serial organization of multiple action units for a proximal task – a reach-to-grasp-and-hold, or a reach-to- grasp-to-eat.	1–3 s
Secondary, social	Immediate, proximal shared project–shared sequence of actions	Interpersonal coordination and serial organization of multiple action units, achieving joint conclusion, for, e.g., reciprocal eye gaze and recognition in mutual attention, e.g., baby looks at mother, mother looks at baby, and smiles	
Tertiary, solitary	Imagined, distal project – projects of projects	Coordination and serial organization of immediate acts as part of a project to complete in the near future, as, for example., a toddler stacking cubes, or playing with grass and flowers, and developing such tasks as an adult cooking dinner.	More than 3 s, typically 5–15 s for single mother-to-infant narratives.
Tertiary, social	Imagined, shared narrative -shared projects of projects	Interpersonal coordination and serial organization of short projects for recall to build experience, for example as in. infant-adult proto-conversation, games, and rituals.	

*See **Table 2** for the intrinsic timing of actions of adults and infants.*

## Ontological Units of Mind-in-Action and the Composition of Embodied Narratives

**FIGURE 1:**
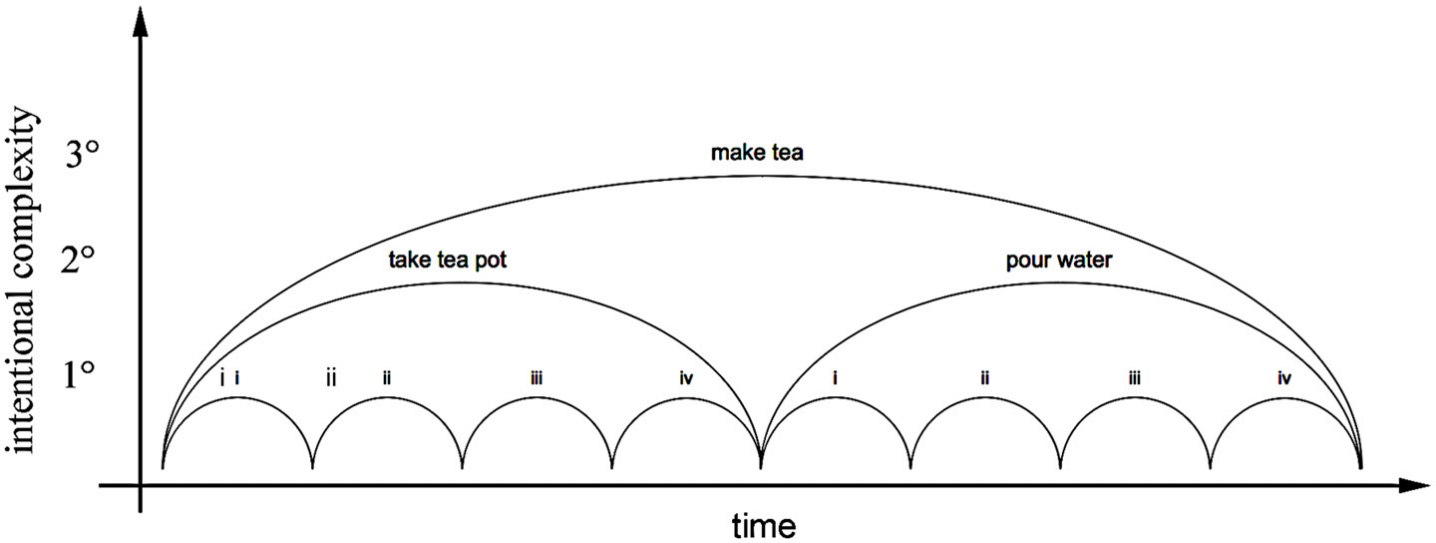
**Hierarchical organization of units of intentional sensori-motor action: (1°) individual ‘action units’ toward immediate goals; (2°) proximal projects that structure and coordinate elementary action units; and (3°) projects of projects.** For example, the distal tertiary intention to ‘make tea’ is accomplished by a sequence of secondary levels of intention; to ‘take tea pot,’ ‘pour water,’ each composed of a sequence of more proximal sensorimotor actions: (i) reach and (ii) grasp the tea pot, (iii) place into position, and (iv) release; (i) reach and (ii) grasp the kettle, and (iii) pour water into the tea pot before (iv) returning the kettle to its resting position. Overlapping projects are possible with use of two limbs or others effectors, with simultaneous action coordinated within a single body for a single practical purpose. Such unitary and embedded organization of practical skill enables a rich repertoire of possible projects. See also Delafield-Butt and Gangopadhyay (2013).

Animal and human movement is integrated in rhythmic and graceful sequences of discreet units of activity, each with their own particular goal-orientation, which are coordinated by the purpose of a higher-order goal or project (Powers, 1973; Condon, 1979; Jeannerod, 1988; Lee, 2009; Trevarthen et al., 2011; Delafield-Butt and Gangopadhyay, 2013) (**Figure 1**). Each unit in the hierarchy of intended action, from unit components to the whole project, comprises both a physical pole and a mental one – the act in motor expression, and its attendant psychological qualities of intention, perceptual guidance, and affective evaluation. These combine in a deliberate form of movement appreciated privately or subjectively, and perceptually available to others intersubjectively (Delafield-Butt, 2014)^1^.

To approach an environmental goal with an independently mobile body many action units must be coordinated together in an intended future (Lashley, 1951; Pezzulo and Castelfranchi, 2009; Pezzulo, 2011; Delafield-Butt and Gangopadhyay, 2013). Serial organization of action and accommodation of new possibilities for further action require more complex cognitive tools of motor planning and associative and episodic memory of a world of opportunities. The cognitive advances in prenatal development, evident before neocortical awareness is functional, are mediated by brainstem and midbrain processes that define a simple action space regulating movements in a time span of approximately 1.2–3 s (**Table 2**). They constitute the beginnings of conceptual development accumulating motor memories and associated responses, affects and plans to achieve the goals of ‘projects of activity.’

**Table 2:** Timing of actions and experience by intrinsic vitality dynamics in adults and infants (Osborne, 2009; Trevarthen, 2009, 2015).

		(A) Somatic, ergo-tropic						(B) Visceral, tropho-tropic		
		Elementary Fast Actions			Conscious sensori-motor control of actions “psychological present”			Imagined and remembered, with emotion		
		5–20	30–40	50–100	150–200	300–700	700–1500	3–6	10–30	30–50
			
		Milliseconds						Seconds		
Adult body and brain	Brain Physiology	Gamma oscillations		Physio-logical tremor	N200 ‘Mismatch’ wave	Memory ‘up-date’. Odd-ball effect	Readiness expectancy-wave	Breathing rhythm	Vaso-motor waves	Heart; para-sympathetic cycles
	Walking		Fast reflex		Step, heal-to-toe	Running, to normal walk	Slow walk			
	Manipulating		Twitches	Fiddling	Finger tapping	Fast reach, to grasp. Hitting	Slow reach Sawing	Movement sequence		
	Eye and head			Eye-saccade	Inter-saccade interval. Head-turn	Separate head orientations		Eye movement ‘scan path’		
	Inspecting objects, reading				Short visual fixation.	Long fixation. Looking response				

Adult communications	Utterances, gestures, expressions				Fast gesture. wink, laugh, spasm, gasp. Patting fast.	Nod. Glance. Eyebrow raise. Hand wave. Laughter burst.	Slow smile. Scowl. Stare.			
	Conversation timing				Overlaps. Interruptions.	Single utterance.	Short turn	Long turn		
	Speech		Voice Onset Time	Fastest lip and tongue articulation	Articles, prefixes. Count fast.	Syllable, vowel. Chewing	Word	Phrase. Breath-cycle.		
	Music			Trills	Vibrato	*presto* to *andante*	*andante* to *largo*	Phrase		
	Poetry				Unstressed syllable	Stressed syllable	Foot	Phrase	Stanza	
	Singing				Vibrato	Beat	Bar	Phrase	Verse	
	Vitality contour				Fast, bursting	Controlled	Slow, graceful	Slow, sedate	Timeless floating	

Infants				Eye saccade.	Blink. Quick head turn. Short ‘coo.’	Inter-saccade gap. Slow head turn. Surge in reach or gesture. Long call, cry.	Pre-reaching lift. Sucking. Beat of proto-conversation. Breathing.	Pause in sucking. Vocal phrase.		

Serial organization of effective movements by an infant, requiring ‘action chaining’ in imaginative projects (Fogassi et al., 2005; Cattaneo et al., 2007) is not well-formed until 9 months after birth. But the newborn infant is able to coordinate whole body movement to identify and track an object of interest, and the basic form of a reach to grasp is already established (Trevarthen, 1984). The goal-directed, prospective control of this ‘pre-reaching’ improves rapidly (von Hofsten and Fazel-Zandy, 1984; von Hofsten, 1991) and by 9 months when the child is sitting upright and the hands are free to manipulate the world, a large number of projects with ‘secondary’ intentionality flourish as the child learns the affordances and delights of the objects around him (Bruner, 1968). At this age the infant develops motives to gain others’ interest by gesture with affective expression, with deliberate attention to the form and direction of their orientations and gestures (Trevarthen, 1986b).

Every purposeful act, at each stage of development, is conceived in both embodied space and embodied time. It is structured by circumstances and directed toward an anticipated future, even those made *in utero*. It must (i) initiate toward that future, (ii) develop in its progression over time and through space with sensory feedback and adaptive anticipatory response charged with memories, and (iii) reach its target before (iv) resolving into a quiet state again, the effects of that action now appropriated into the recollected state of the organism. Each step carries the organism purposefully in time and space to a set of relations with new affordances, and new meaning in what Margaret Donaldson calls the ‘line mode’ of thought (Donaldson, 1992).

Within the hierarchy of action organization (**Figure 1**; **Table 1**; Delafield-Butt and Gangopadhyay, 2013), each level is organized by its local, prospective goal, in coordination with levels above and below. The simple ‘action unit’ serves as the basic element of intentional action, characterized by continuous regulation of velocity to reach an anticipated future state in self-related space and time. Hand gesture, pressure change in grasping, oral movement in speech, a step in walking, etc. are each defined as closure of an ‘action gap’ (Lee, 2009). Such simple action units are ‘goal-directed’ and seek closure. They occur first in early fetal stages and constitute the *primary* level of sensorimotor intentionality. In purposeful behavior by a more developed conscious subject, a sequence of these elements can be serially organized into a *secondary* level of a ‘project’ of action units (Lashley, 1951). A reach-and-grasp or a reach-to-touch is the first rudimentary project of a forelimb of a fetus. It is composed of two action units sequentially organized to form one coherent project with a common goal.

### Innate Micro-Kinesics of Communication, and Emotional Regulation of Projects and Stories

Measurement of motor activities to small fractions of a second by ‘micro-kinesics’ has demonstrated both the fine coordination of movements within an individual preforming an activity of tool use, or of locomotion (Bernstein, 1967), and the delicate inter-synchrony of utterances and gestures between individuals in natural conversation or artful performance (Condon and Ogston, 1966; Birdwhistell, 1970). This has been applied to prove the sensitivity of a newborn to the motor impulses of adult speech – the baby can move its arms to synchronize precisely with the syllables and phrases, which anticipates learning to speak (Condon and Sander, 1974; Condon, 1979).

As sensorimotor action planning with cognitive capacities is mastered in infancy through primary and secondary levels, the world becomes a place to play with chains of purposes to build memorable projects. The sequential organization of projects develops in toddlerhood to enable *tertiary* ‘projects of projects’ that perform complex and abstract tasks with goals beyond the present moment in both time and action space (Trevarthen et al., 2011).

We can trace the development in the first year of infancy toward projects of practical communication: from primary intersubjectivity in dialog, through games with expressions, then games with objects, to secondary intersubjectivity to share the project of a task using objects (Hubley and Trevarthen, 1979). Halliday (1978) describes stages in the child’s developing powers of conversation with increasingly complex conventions of practice: proto-conversation (with expressive ‘pre-speech’), proto-language (acts of meaning with content), proto-narrative and dialog (with elementary lexico-grammar), proto-discourse (intermediate lexico-grammar), proto-turn taking (advanced lexico-grammar), proto-variation (register and social dialect). The child’s ‘locus of concern’ in awareness of intentions is growing with increasing memory, always regulated by feelings of value and expressed with emotion (Donaldson, 1992). Each level in prospective control of action is structured within the intentions specified at higher levels, and conversely the intentional organization of lower level states motivates and structures those of higher levels (**Figure 1**; **Table 1**).

Each stage of the development of conscious control of actions is sustained by the affective regulation of ‘vitality dynamics’ in body movement (Stern, 2010). These arise as sub-neocortical ‘primary-process emotions’ (Panksepp, 2011) acting with ‘anoetic’ consciousness (Vandekerckhove and Panksepp, 2011), becoming ‘secondary-process emotions,’ learned and supported with basal ganglia memories and associations of moving, then ‘tertiary affects,’ enabled through neocortical awareness of richer environmental affordances. Maturation of core brain systems motivates development from primary sensorimotor units of ‘intention-actions’ to tertiary, abstract projects of ‘intentions-to-act’ in ‘rational’ ways, each stage being prospectively controlled to coordinate and direct action in the ‘specious present’ (James, 1890) to achieve the goal in mind (Pezzulo and Castelfranchi, 2009; Pezzulo, 2011; Delafield-Butt and Gangopadhyay, 2013). Progress from pre-conceptual to conceptual cognition elaborates personal phenomenal awareness from the start of life as the basis for social collaboration (Tomasello et al., 2005), and education of cultural intelligence (Trevarthen et al., 2014; Trevarthen and Delafield-Butt, 2015).

Performance of more distal, more ambitious goals requires serial organization of movement-with-awareness into ‘projects of projects of action units.’ Enterprises like cooking dinner, building a tower of blocks, or winning at chess, require coordinating a number of sub-projects, each with their own goals. Such achievements of creative practical and social action must be conceived within an imaginative ‘present moment’ drawing on memories of past skills as it looks to achieve a desired future (Stern, 2004).

When babies become toddlers and are able to master a rich repertoire of projects, they take delight in mimicking the complex projects of adults, learning the styles and patterns of a culture. Cooking dinner is one such project, and the children’s play kitchen is always a favorite in the nursery classroom. Nursery children aged 2–4 take special joy in cooking and preparing dinner with toy utensils and foods, organizing the projects of grasping, cutting, mixing, and so on into larger structures with higher intentional perspective. Over the next years their motor and cognitive precision will improve to allow them to do just that, replacing toys for the genuine article. From two to five the child takes up and transforms words to recount ingenious understanding of purposes and experiences (Chukovsky, 1968).

### Social Learning, from Narratives of Communicative Musicality to Language

Young children join in the rituals of the day (Frank and Trevarthen, 2012), showing special interest in social engagements that incorporate the rhythms and song of dance and music, even after they go to school for more formal instruction (Erickson, 2009). Bruner (1996, p. ix) remarks that “…schooling is only one small part of how a culture inducts the young into its canonical ways.” Social learning is innate, embodied experience shared naturally in cultures of families and community animated by the ‘human sense’ (Donaldson, 1978) of the ‘the muse within’ (Bjørkvold, 1992), its narratives regulated with hierarchical rhythmic structures of moving emotionally with ‘communicative musicality’ (Malloch, 1999; Malloch and Trevarthen, 2009; Mazokopaki and Kugiumutzakis, 2009; Panksepp and Trevarthen, 2009), communicating emotional feelings of the self (Stern, 1993, 2000).

“Narratives of individual experience and of companionship are built from the units of pulse and quality found in the jointly created gestures of vocalizations and bodily movement. Narratives are the very essence of human companionship and communication. Narratives allow two persons to share a sense of passing time, and to create and share the emotional envelopes that evolve through this shared time. They express innate motives for sharing emotion and experience with other persons and for creating meaning in joint activity with others”

(Malloch, 1999, p. 45).

Protoconversations and baby songs from many different languages all show the four-part organization in dialogs, verses or stanzas of between 20 and 50 s in length, with modulation of rhythms and expression to compose *introduction, development, climax*, and *resolution*, with rhyming vowels at key points, to all of which the infants engage with anticipation (Malloch, 1999; Trevarthen, 1999, 2008; Powers and Trevarthen, 2009).

A human full term newborn, adapted for new experiences in a human community, is sensitively responsive to the dynamic impulses of another person – by body contact, sight of the eyes face and hands, the sounds of speech, and gentle touching, and he or she can recognize the mother’s voice (Condon and Sander, 1974; Brazelton, 1979; Nagy, 2011). If calm and alert after the transition to a very different environment, the newborn may contribute to a precisely timed imitative exchange of expressions and pauses with another person, a dialog of movements that evolves as an emotional event lasting a few seconds (Trevarthen, 1999; Kugiumutzakis and Trevarthen, 2015). It is clear that newborn infants hear the beat of human life in movement (Winkler et al., 2009). They move to the rhythms of music (Zentner and Eerola, 2010), co-regulating their impulses in poetic episodes of shared experience.

**FIGURE 2:**
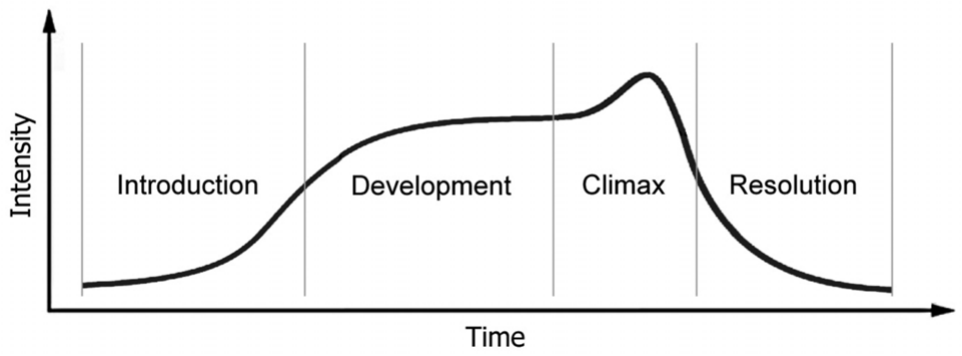
**Narrative intensity contour of impulses to move in the time of a narrative over its four phases.** (i) ‘Interest’ in the narrative begins at a low-intensity in the *introduction*, which ‘invites’ participation in purposefulness; (ii) the coordination of the actions and interests of real and imagined agents intensifies over the *development*, as the ‘plan’ or ‘project’ is developed; (iii) a peak of excitation with achievement of a goal in mutual intention is reached at the *climax*; after which (iv) the intensity reduces as the purposes of the participants share a *resolution*, and those who were closely engaged, separate. From Trevarthen and Delafield-Butt (2013a).

Human exchanges of purposes and feelings are mediated by motor signals of a complexity not possessed by any other primates – of the head, the eyes, the face, the vocal system, the hands, and the whole body, all active in well-ordered sequences from birth, and all conspicuously shaped to make signals that another human being will appreciate (Trevarthen, 1978, 2012). The movements are synchronized to express a contour of energy showing of a ‘regulatory tide of vitality’ (**Figure 2**), the rhythm of which relates to autonomic or visceral processes that give the internal ‘psychic time’ (**Table 2**) of an integrated Self to arousal of sequences of cognitive elements, intentional acts, and the affective, interpersonal power of expressions in communication (Delamont et al., 1999; Keltner, 2003; Wittmann, 2009; Meissner and Wittmann, 2011). The movements are intricately ordered with prospective sensory control common to all organisms (Trevarthen et al., 2011; Delafield-Butt et al., 2012).

Within the first 2 months, developments in the alertness and focus of the infant’s attention to human signals, especially for finding and keeping eye-to-eye contact and precise synchrony with vocal and manual expressions, encourages a parent to share dialogs described as ‘proto-conversations’ (Bateson, 1979). Expressions of intention, awareness, and feelings are passed between infant and parent in ‘proto-narrative envelopes’ of vitality (Stern, 1999, 2010), with an evolving shape as celebrations in gesture and vocalization (Trevarthen, 1980, 1990). Groups of infants less than 1 month old with no adults present can indulge in similar dramas of vocal and gestural display (Bradley, 2009). These are semiotic events of imaginative movement, which, months later, develop into stories told in the melodies of song (Bjørkvold, 1992; Malloch and Trevarthen, 2009), and elaborate forms of language (Halliday, 1978; Rommetveit, 1998). We are born to be ‘story-making creatures’ (Bruner, 1990). And stories are born of the motor logic of agent movements seeking vital purpose (Delafield-Butt and Trevarthen, 2013).

Episodes of dramatic or more aroused action, of speaking in conversation, or of music, appear to be essential in the emotional regulation of all forms of movement (Damasio, 1999) and to all forms of inter-subjective co-creation of meaning in dyadic states of human consciousness (Trevarthen, 2005; Tronick, 2005). They make predictable patterns of engagement, and lead to mutual ‘sympathetic’ involvement in vocal and motor expressions of changes in feeling. As Adam Smith said of music in his remarkable essay on *The Imitative Arts*: “Time and measure are to instrumental Music what order and method are to discourse; they break it into proper parts and divisions, by which we are enabled both to remember better what has gone before, and frequently to foresee somewhat of what is to come after:.... the enjoyment of Music arises partly from memory and partly from foresight.” (Smith, 1777/1982, p. 204). Imagined worlds of art and reason, with their emotional appreciation, are built out of the times experienced in familiar episodes of expressive behavior, the vitality contours of which may be anticipated, recalled, and shared with companions.

We trace the intensity contour of energy in movement over four phases of a message or shared performance: in the introduction attentive expressions ‘invite’ participation in purposefulness; the first response from a real or imagined partner provokes the development of the ‘project,’ until a peak of coincident excitation in mutual intention is reached at the climax, after which the intensity of expectation and effort reduces in a resolution. Then those who were closely engaged, separate, or engage in a new narrative process, co-creating new meanings and revisiting old ones. The pattern of rise and fall in excitement and effort may also be found in the semiotic rituals of animals (Tinbergen, 1951; Sebeok, 1972, 1994).

A narrative’s nature exists in its internal dimensions of feeling and form as well as in its rhythmic, shared, and co-created form between two or more persons, “There are certain aspects of the so-called ‘inner life’—physical or mental—which have formal properties similar to those of music— patterns of motion and rest, of tension and release, of agreement and disagreement, preparation, fulfillment, excitation, sudden change, etc.,” (Langer, 1942, p. 228, quoted by Kühl, 2007, p. 223). Co-created narrative engagement gives structure to intersubjective episodes making discreet parcels of interaction with definitive opening and conclusions, as a solo sensorimotor project does. Its musical nature further functions to “enhance… the quality of individual experience and human relationships; its structures are reflections of patterns of human relations” (Blacking, 1969/1995, p. 31). The musicality of narrative “is inseparable from its value as expressions of human experience.” (*ibid.*).

When the narrative is finished, the experience of its creation will remain with each of the partners, and between them they may hold its special memory – a memory of a unique, shared experience, the co-creation of which imbues the memory with ‘meaning.’ The conclusion of a narrative episode is followed by a disengagement, which allows the two partners to consider renewing their mutual focus, ready to begin building a new narrative cycle, or they may separate. Read and Miller (1995, p. 143), social psychologists, consider narratives to be “universally basic to conversation and meaning making.” They can be regarded as the essential foundation for consciousness in a more elaborate purposeful social life, among animals, for infants, and for older human beings who have mastered language (Dautenhahn, 2002). Narratives do not have to be linguistic. Understanding the pre-verbal origins of narrative is fundamental for understanding human cognition and culture, and demands multidisciplinary investigation (Bullowa, 1979; Cobley, 2014).

## The Life Time of an Early Embodied Narrative: an Intimate Illustration

We illustrate the primary psychological events and their synthesis to generate mutual interest, shared excitement, and reflective satisfaction with a micro-analysis of a 30 second dialog between a premature newborn and his affectionate mother. Many forms of expressive movement are displayed, intersubjective contact being mediated by complementary modalities of voice and gesture. The two human beings compensate for their very different levels of development in intimate collaboration, the mother coming close to her infant in rhythm and sympathy with musical intonation of her speech, and the infant animating his feelings in seductive ways in response to her encouragements.

Baby B, was born prematurely at 27 weeks gestational age with his monozygotic twin brother. The boys received intensive care in the hospital neonatal unit, and their mother visited every day for sessions of body to body ‘kangaroo’ care and for social support. When the recording was made, the mother and her infants had been in hospital for 8 weeks with regular opportunity for interaction, and B was at 34–36 weeks before term. Both babies were due for discharge that week and their health was considered stable.

**FIGURE 3:**
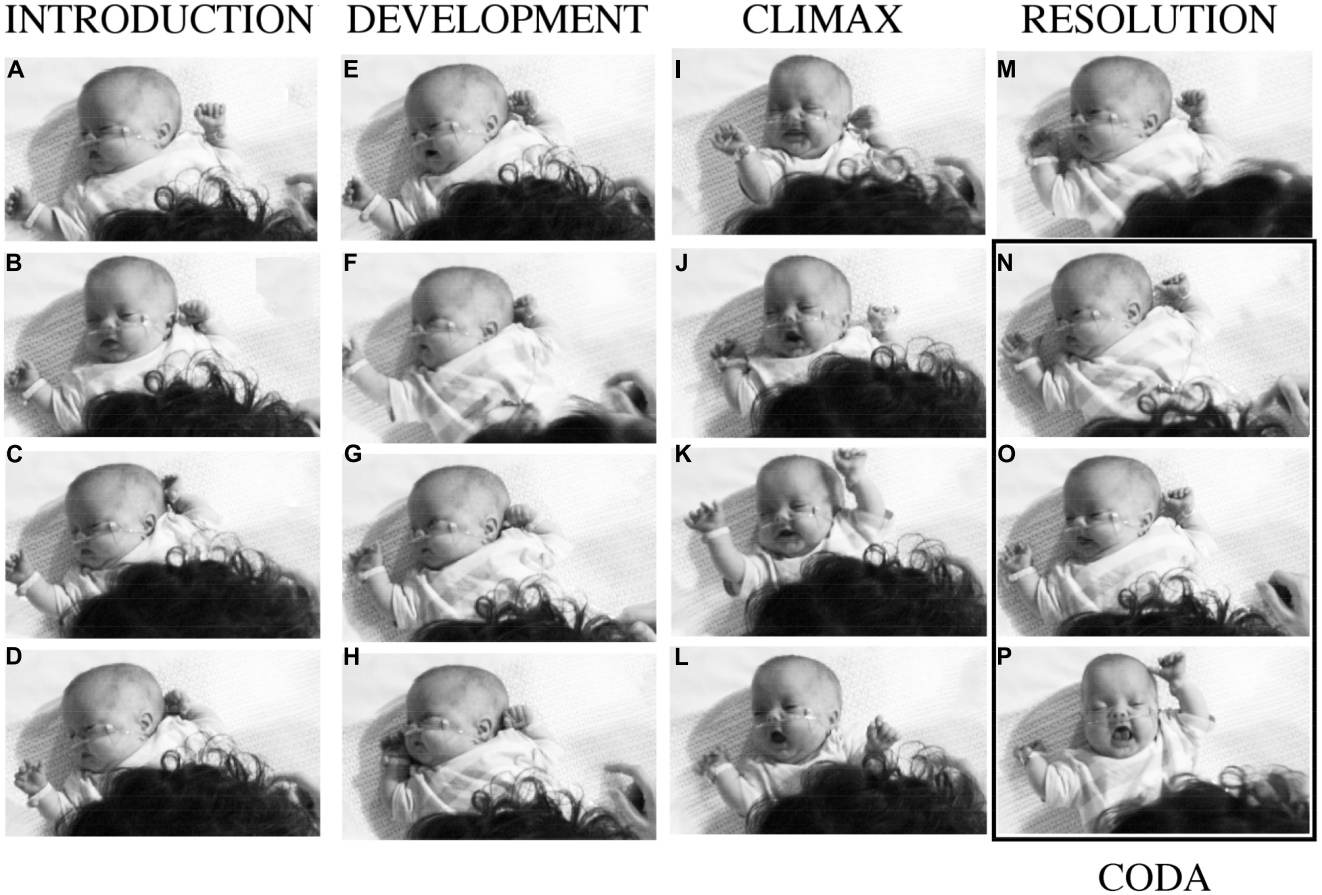
**Baby B’s expressive actions at different moments in interaction with his mother.** The columns correspond to the four phases of a narrative of purposes and experiences supported by the vocalizations of his mother numbered in **Figure 4** After a period of intense, pleasurable self-expression, with his mother’s happy participation, B withdraws, and his mother attempts to provoke new engagement by ‘teasing’ or ‘joking’ about his behaviors. See detailed description in the text.

**FIGURE 4:**
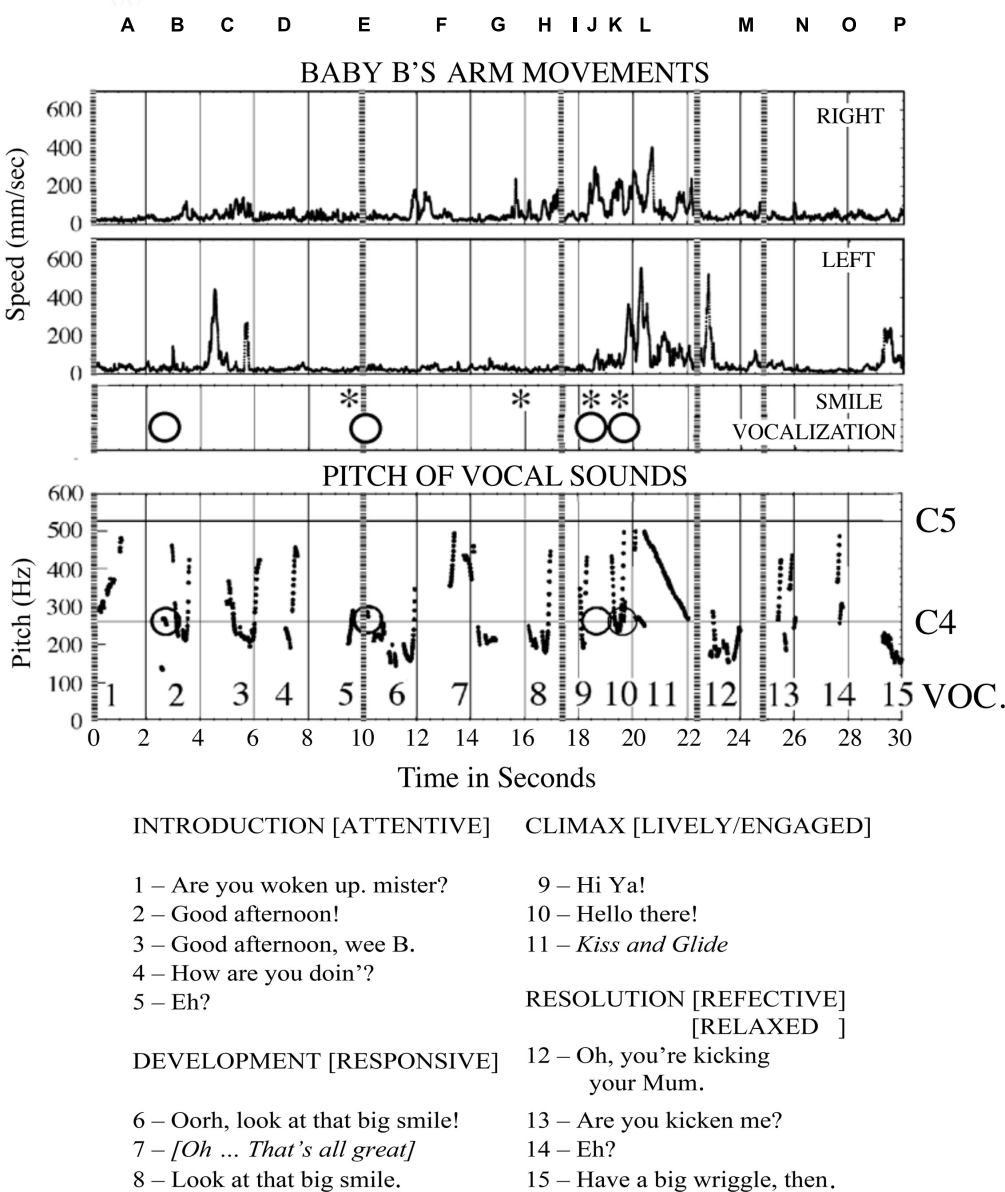
**Sounds recorded through the 30 s dialog with Baby B, and his arm movements, smiles and attempts to vocalize.** Along the top, the locations of the photographs shown in **Figure 3** are shown by letters A–P. Below is a transcription of the mother’s speech and vocal expression. The utterances are numbered and identified in the pitch plot above. The baby’s vocalisations are indicated with a circle, they do not always register on the pitch plot. Photographs N, O, and P, and utterances 13, 14, and 15 cover the final period when the infant is not engaged with his mother’s expressions, and her speech indicates she is provoking him, or joking about his actions. He repeats the arm movements he made at the ‘climax,’ as a ‘coda.’

B was lying on a quilted tabletop with his mother seated in a chair at his feet, leaning over him. An overhead video camera provided a vertical view of B (**Figure 3**), and a second camera recorded a frontal view of his mother. Their vocalizations (**Figure 4**) were recorded by two microphones. B’s arm movements were tracked by attaching a reflective marker to his wrists. A six-camera motion capture system (Proreflex 500, Qualisys) recorded in 3D the coordinate position of the markers 500 times per second, with a spatial resolution of less than 1 mm. Displacements of his left and right wrists were recorded as tangential velocity or speed, disregarding direction of travel (**Figure 4**).

B was intubated from birth to assist his breathing, which made vocalizations difficult. When recorded he was oxygenated by a nasal cannula. Medical physiological measures were taken continuously to monitor heart rate and blood oxygenation. A warning sound alerted his mother when these levels dropped below thresholds set on the monitor standing to her right and behind her.

At first, B was sleeping quietly, and his mother was asked to try to wake him and to keep him alert, but calm. The 30 s sample of their communication, starting from the moment he began to respond, illustrates how, by sympathetic attention and affectionate speech, she invited and responded to B in intimate dialog, building from his active expressions of attention to her a narrative episode of engagement. She was following his expressions of interest or enthusiasm, and it may be said that B was the main ‘composer’ or ‘author’ of this story, animating its beginning and drawing it to conclusion with his actions. The mother invited him then followed his animation and willingness ‘play the game’ with her. Their mutual interest in the ‘dialog’ was sustained by the ‘attunement’ of the rhythmic vocal invitations of the mother (Stern, 2000), with occasional touches by her gesturing right hand, and by the eagerness of the infant to respond with expressive body movements, hand gestures, attentive looking, smiles, and vocalizations (Trevarthen, 1990). The photographs in **Figure 3** record events in the infant’s participation with certain of the mother’s expressions. They demonstrate the precise inter-synchrony of their complex behaviors. The mother’s speech and non-verbal vocal expressions and B’s arm movements, smiles and attempts at vocalization are shown in **Figure 4**. Photographs of **Figure 3** are indicated by their letters, and the mother’s utterances are printed in italics.

### Introduction, 0–9 s

In the first 3 s (A,B) the baby, who had been asleep, is stirring gently as his mother attempts to arouse him, saying, “*Are you woken up Mister?*” He turns sleepily with eyes closed, to face her (B). His left hand is held shut by his head and his right hand is lying back on the bed, open. He makes a small vocalization just before she says, “*Good afternoon!*” She makes a gesture to touch his side with her right index finger then points to her mouth in rhythmic synchrony with speech before laying her hand gently on the bed beside him. B closes his mouth with his tongue between his lips, then turns away, making a sleepy jerk to face his right hand, with eyes closed. His left hand makes a rapid movement up and to the right to follow the head, and his right hand moves up a little the fingers opening as his mother starts to say, “*Good afternoon, wee B*” and she turns left to face him, placing her right index beside B. His right hand opens and shuts synchronously with the word “B” – he is listening (C). At 7 s as his mother says “*How are you doin?”*, B’s right hand moves slightly forward, closes index then opens and closes again gently, again in synchrony with her speech (D). She withdraws the right index as she says “*Eh?*”, as her voice rises, B smiles and his right hand shuts. He is clearly listening and shadowing his mother’s speaking with his right hand, and signaling his appreciation with a smile.

### Development, 10–17 s

Still smiling, B makes a small vocalization at 10 s (E) His mouth opens and then shuts quickly as his mother says “*Oorh, look at that big smile!*” with a cheerful teasing expression of pleasure, and she pulls her head back and touches the bed with her right index. B may be swallowing after the attempt to vocalize. After smiling, he pauses a moment. His mother turns right to look at the monitor behind her to check his physiological indices and says, to herself, with different intonation, “*Oh, that’s all great!*” B reaches up with his right hand and opens his eyes in synchrony with this phrase (F), then as she pronounces the word “*great*” he makes a chewing movement and looks toward his hand. At 15 s his right hand opens and closes and his wrist turns out away from his face (G). He may be feeling the mattress with the back of his hand. His mother touches him to tickle his thigh gently, and then as she says to him, with lift of intonation, “*Look at that big smile!*” her index goes down to touch the blanket. B’s right hand waves back to touch the bed, then is pulled in his cheek. He smiles and his eyes close in rhythm with the mother’s speech, then they open at 17 s (H).

### Climax, 18–22 s

As B’s mother says “*Hi-ya*,” he stretches his head up to look forward, turning his right hand back at the wrist in a waving gesture, opens his mouth wide, and smiles (I). Then he turns quickly to face his mother, vocalizes with a rough sound and gestures, smiling and grimacing with the effort. At 19–20 s when his mother says, “*Hello there!*”, both his hands are pulled up, back and out in a big rowing gesture (J,K). As mother’s voice glides dramatically down through an octave from C5 to C4 between 20 and 21 s, B opens his mouth very wide and closes it with a smile in synchrony with fast forward and back down and pulled back rowing movements of his two hands. His eyes open and he coughs at 20 s (L). At 22 s B makes a second rowing cycle and his mouth shuts. As his mother finishes the vocal glide and turns to her right, his hands are at his shoulders and his tongue is visible in an open mouth.

### Resolution, 23–30 s

At 23 s, having finished his display of excitement and delight, shared intimately in rhythm with his mother’s speaking, B pulls his hands in and turns away from his mother, eyes half closed, mouth half open and shutting with tongue visible as she says with a deep ironic tone “*Oh, you’re kicking your Mum!*” (B is pushing his foot against his mother’s stomach). He turns his head up, and with the word “*Mum!*” (M), his eyes close and his right hand goes up, opens and moves slowly out, then his eyes open to look at his hand. His mouth is half open with the tongue visible. His mother glances to her right to the monitor as she says “*Mum!*”, then she turns back and gives a little tickle to his side with her right index, saying, “*Are you kickin’ me?*” (N). At 26 s B clasps his right hand quietly and moves it down as his mouth opens. His mother says, “*Eh?*” (O). A moment later, at 29 s, B turns back to face his mother, his mouth opens wide and his eyes close. His left hand is up beside his face and his right hand is back and half closed, with index extended and his tongue protruded in a wide-open mouth (P). His mother responds with a vigorous teasing at low pitch, “*Have a big wriggle, then.*” In these last 5 s (N,O,P) B has become active, but he is performing with little sign of awareness of his mother, or her playful actions. He appears to be disengaged from her and perhaps is recollecting his excitement of 10 s previously (I,J,K,L) in a ‘coda.’ He does not smile or vocalize.

### Summary

B displayed his awakening experience of his mother’s voice and touches by becoming still, to listen. And then he slowly began a story of self-aware movements that harmonized with her repeating calls of speech, turning his head, gesturing expressively with his hands, making subtle movements with his mouth, holding his right hand up and looking at it, recognizing his mother’s encouragements with smiles and attempts to vocalize. He became a collaborator, linking his increasing interest and effort at self-expression to his mother’s responses, gaining fluency and vigor in synchrony with her rhythms until he excited both of them with big cyclic movements of both arms together and wide open mouth with effort to vocalize. His mother’s dramatic pitch glide shared this triumph and brought their shared excitement to a close. And then he withdrew and rested before making some movements for his own pleasure, which show little attention to his mother’s observations about what he was doing. Altogether they traced a narrative form of arousal, affect, and intention with its four distinct phases, co-creating its ‘story.’

### Generating Narrative Patterns of Meaning in Learning and Memory

B’s shared narrative with his mother appears to have become an object in his memory, holding a process of learned meaning composed with evolving affective value and patterns of self- and other-regulation of arousal, interest, and intentions expressed in the form and qualities of body movement. He repeats this pattern in the short coda after the event, recalling the embodied, shared schema of feeling in action and expression. This object now held in his memory becomes available for future events, setting a template of possible action with its expectations of affect-laden social interest and intention from the others, as well as its preparatory autonomic anticipations (Schore, 2000). This is how his attachment with his mother, and their shared, intimate understanding, grows (Powers and Trevarthen, 2009; Porges and Furman, 2011; Narvaez et al., 2013).

## Human Being in Movement and the Making of Meaning, in Learning, and in Therapy

The story made by Baby B and his mother demonstrates the dual aspect of narrative as embodied in physical and emotional experience of the actor, and as a semiotic experience for consensual understanding. Mother and baby shared a human ‘tide of vitality’ in arousal, interest and expression over the four-part structure of what Stern (1995) calls a ‘proto-narrative envelope.’ The mother elaborates this in a verbal story-making, making sense in her own language of the rise and fall of vital interest and pleasurable feeling they share. Together, their bodies give form and energy to a tide of meaning sensed in many modalities, on which her verbal language can ride.

All narratives are rooted and expressed in body movement, for communication. Human cultural narratives, beginning in proto-conversations and the rituals of games with infants (Merker, 2009), become habitual sources of collaborative activity between people who know each other well. In enduring relationships productive interactions pick up themes from earlier narratives, thus developing a memory or ‘habitus’ of engagement that builds cultural meanings of ritual and belief, in art and industry in a ‘sociosphere’ of knowledge (Bourdieu, 1990; Frank and Trevarthen, 2012). The ritualized games enjoyed between an infant and a mother or other loving companion strengthen their affection and give them a sense of meaningful ‘belonging.’ They create a ‘proto-habitus,’ an early ‘living in belonging’ (Gratier and Trevarthen, 2008).

The theory of the prospective organization of embodied narrative in interpersonal meaning-making is supported by evidence of disruption in the prospective timing and affective integration of motor intentions in individuals with socio-emotional disorder. For example, errors in sensorimotor capacity to efficiently enact desired intentions characterize autism spectrum disorders, regularly thwarting success, creating distress and isolation, and consequent social and emotional compensations (Trevarthen and Aitken, 2001; Trevarthen et al., 2006; St Claire et al., 2007; Trevarthen and Delafield-Butt, 2013b). When rhythms of shared narrative become disrupted, causing social misunderstanding and anxious or defensive reactions, therapy for emotional illness or autism can benefit from understanding of core intentional and affective dynamics and their regulation by sharing imitative and creative projects, which may employ non-verbal forms of expressive movement as in dance, music, and drama.

## Conclusion

In this paper we trace the origin of narrative form in communication to a primary motivation for conscious understanding enacted and structured through purposeful movement. These lively self-generated engagements with awareness of the world place events in a time and space of vital meaning for the embodied experience of the Self, as well as for communication with other persons. The form of this generative process is found to be invariant across development, from the simple motor capacities of the young fetus through pre-verbal proto-conversation in infancy, and into linguistic meaning-making in childhood and later human life.

Meaning-making in movement and its co-creation in dialog arise within a basic, four-part organization common to all levels in the embodied action of a purposeful agent, with (i) an *initiation* seeking a goal, (ii) a *development* in the strategy of its progression, usually through repeated cycles of ‘testing’ by expression and adaptation until, (iii) a *climax* of excitation and achievement is attained, before (iv) that particular plan of action comes to an end, or *resolution*, and the direction of interest changes.

We have shown how the future-oriented, generative and rhythmic structure of the human will-to-move is organized from the start by the psycho-motor dynamics of an articulated, hypermobile body together with its internal self-preserving, visceral autonomic rhythms, that is, with purpose and with emotion. The coordinated temporal patterns of action and vitality of the Self-As-Agent structure forms of effort that generate meaningful concern for the individual as he or she, in solitary or in social projects patterned over many seconds, tens of seconds, minutes, and hours, engages with a world of objects and people. The rhythms of engagement expand in scope and ambition as the imagination of adult life envisages years of achievement and understanding that will be stored and named as beliefs, rituals, and techniques. Thus life stories with their intrinsic narrative vitality create a store of experience, memories, understanding and purpose – the culture of a cooperative society.

## Conflict of Interest Statement

The authors declare that the research was conducted in the absence of any commercial or financial relationships that could be construed as a potential conflict of interest.

## Footnotes

1 We view consciousness as realized by a purposeful composition of elementary intentions with knowledge of their individual and collective consequences, chained into the future with some precision. In defining conscious experience, James (1890) proposed that awareness, although also apparently fluid and continuous, is fundamentally atomistic (Sinclair, 2009). The post-Cartesian process ontology of Whitehead (1929), his “Philosophy of Organism,” helps to make sense of the agentive, goal-oriented nature of animal action and offers a description of the mind-body relationship better suited to the nature of embodied cognition as participatory and generative meaning-making than Platonic or Cartesian accounts (Delafield-Butt, 2007, 2014; Basile, 2010) with their explanatory gap (Chalmers, 1995). At the same time, it is compatible with a robust, mechanistic materialism suitable for scientific enquiry (Strawson, 2006). Langer (1942), following Whitehead, advanced an artful philosophy, drawing support from the persuasive power of music. Also concerned with the embodied and inter-subjective nature of experience, and profoundly relevant for our account of the developing human mind is the phenomenology of Merleau-Ponty (Welsh, 2013).
